# Laboratory assays on the effects of a novel acaricide, SYP-9625 on *Tetranychus cinnabarinus* (Boisduval) and its natural enemy, *Neoseiulus californicus* (McGregor)

**DOI:** 10.1371/journal.pone.0199269

**Published:** 2018-11-05

**Authors:** Jingqi Ouyang, Yajing Tian, Chunxian Jiang, Qunfang Yang, Haijian Wang, Qing Li

**Affiliations:** Department of Plant Protection, College of Agronomy, Sichuan Agricultural University, Chengdu, Sichuan, P. R. China; Chinese Academy of Agricultural Sciences Institute of Plant Protection, CHINA

## Abstract

**Objective:**

*Tetranychus cinnabarinus* (Boisduval) is an agricultural mite pest threatens crops throughout the world, causing serious economic loses. Exploring the effects of acaricides on predatory mites is crucial for the combination of biological and chemical control of *T*. *cinnabarinus*. *Neoseiulus californicus* (McGregor) is one of the principal natural enemies of *T*. *cinnabarinus*, which can be applied in protected agriculture. In this study, the effects of sublethal concentrations of a new acaricide, SYP-9625 on two mite species, and the effects of the application concentration on predatory mite, *N*. *californicus* were assessed. The aim of the present study was to evaluate the effect of SYP-9625 on life parameters and predation capacity of *N*. *californicus* based on the concentration-response bioassay of *T*. *cinnabarinus* to explor the application of the new acaricide with natural enemy *N*. *californicus*.

**Method:**

All of the experiments were conducted under laboratory conditions [25 ± 1°C, 16: 8 h (L: D) and 75 ± 5% RH]. The sublethal concentrations LC_10_ (0.375μg/mL) and the LC_30_ (0.841μg/mL) against *T*. *cinnabarinus* and the application concentration (100μg/mL) against *N*. *californicus* were used to evaluate the effects of SYP-9625 on population parameters of *N*. *californicus* based on an age-stage, two-sex life table and its predation capacity by functional response.

**Result:**

*cinnabarinus* females treated with LC_30_ exhibited significantly reduced net reproductive rates (*R*_*0*_ = 11.02) in their offspring compared with females treated with LC_10_ (*R*_*0*_ = 14.96) and untreated females (*R*_*0*_ = 32.74). However, the intrinsic rate of increase (*r*_*m*_) and the finite rate of increase (*λ*) of *N*. *californicus* indicated that the application concentration of SYP-9625 had no significant negative effect on *N*. *californicus* eggs (*r*_*m*_ = 0.277, *λ* = 1.319) compared to the control (*r*_*m*_ = 0.292, *λ* = 1.338). Additionally, most population parameters of *N*. *californicus* showed a dose-dependent manner with the increase of the concentration of SYP-9625 against *T*. *cinnabarinus*. SYP-9625 also stimulated the control efficiency of *N*. *californicus* against immobile stages including eggs and larvae.

**Conclusion:**

This study demonstrated that sublethal concentrations of SYP-9625 can inhibit the population growth of *T*. *cinnabarinus*. In addition, the sublethal concentrations and the application concentration showed no effect on the population growth of *N*. *californicus*. These two advantages described above showed great commercial potential of this new acaricide based on population parameters of the two mite species and predation capacity of the predatory mite under laboratory conditions.

## Introduction

Nowadays, agricultural spider mite pests are becoming one of the major threats to some important crops such as vegetables, fruits and ornamentals throughout the world. Most spider mite pests, such as *Tetranychus cinnabarinus* (Boisduval), have gained rapid resistance resulting from frequent applications of acaricides [1, 2]. Therefore, new acaricides with excellent insecticidal activity and low toxicity to natural enemies are becoming necessary [3].

In integrated pest management (IPM) systems, natural enemies and compatible acaricides can be applied in a conjunct group, and a proportion of studies tend to paying more attention to the toxicity of acaricides on predatory mites [3–7]. Based on the inter-population differences in the sensitivities of these natural enemies, Lima evaluated different acaricide toxicities against *Neoseiulus barkeri* (Hughes) and suggested that fenpyroximate and chlorfenapyr can be used together with the predatory mite application [4, 8]. Recently, the sublethal effects of acaricides have been considered a more accurate approach to measure toxicity than direct contact toxicity [9]. Mollaloo investigated the effect of three lethal concentrations pyridaben on the developmental and reproductive parameters of *Neoseiulus californicus* (McGregor), which confirmed that the maneuverability about the combination of natural enemies such as phytoseiid predators with compatible acaricides is the key to decrease not only chemical applications but also the environmental hazards [10]. Furthermore, pest suppression by a predator species strongly depends on two major components of predator-prey interactions: the predators’ numerical and functional responses [11, 12]. Pesticide exposure can significantly influence the functional response of predators, so many studies have assessed the effects of pesticides on the functional response of predatory mite species [13]. For example, Poletti reported that although acetamiprid did not affect the functional responses of *N*. *californicus*, it weakened the predation capacity of *Phytoseiulus macropilis* (Banks) [4].

The predatory mite *N*. *californicus* is one of the principal natural enemies of tetranychid mites in several countries andpromotes the efficient control of those mites in several crops [8]. Moreover, *N*. *californicus* exhibits broad environmental tolerance, and is used to manage pest mites in many countries, thus demonstrating the great biological control potential of *N*. *californicus* [14–17].

SYP-9625 is a new acaricide which has been registered as the commercial formulation, TC 98% in China. It is one of a series of novel pyrazolyl acrylonitrile derivatives that has shown excellent acaricidal activity against *T*. *cinnabarinus* and very low toxicity to mammals [2]. Before promoting this new acaricide, it is important to evaluate its effects of applying on the pest mites as well as the natural enemy *N*. *californicus* under laboratory conditions to determine the reasonable concentration of SYP-9625 that has excellent insecticidal activity and low toxicity to *N*. *californicus* is also crucial. This study investigated the sublethal effects of the new acaricide SYP-9625 on two mite species and the effects of the application concentration on population parameters of *N*. *californicus* based on the two-sex age specific life tables. The functional response of *N*. *californicus* exposed to SYP-9625 was also assessed to evaluate its predation capacity.

## Materials and methods

### Insect cultures

The *N*. *californicus* colony was originally sampled in Sichuan Province, China in 2010 and was reared on detached kidney bean plants (*Phaseolus coccineus* L.) infested with *T*. *cinnabarinus* in the laboratory conditions. The *T*. *cinnabarinus* colony was collected from a farm located at Sichuan Agricultural University, China. Glass petri dishes (9 cm in diameter) were used to construct rearing arenas that were sealed using plastic wrap. A thin cotton layer was placed at the bottom of the Petri dish, and an upturned bean leaf was placed on the saturated cotton and surrounded with water to prevent the escape of the mites. The kidney bean leaves were replaced every week. All tests were conducted in the laboratory at a photoperiod of 16: 8 h (L: D), 25 ± 1°C and 75 ± 5% RH [3].

### Chemical tested

SYP-9625 is a new acaricide that has been registered as the commercial formulation, TC 98% in China. It was synthesized by Yu et al., to target *T*. *cinnabarinus* and was obtained from Shenyang Sinochem Agrochemicals R & D Company, Ltd. SYP-9625 is one of a series of novel pyrazolyl acrylonitrile derivatives under an international patent that names a pyrazolyl acrylonitrile compounds and uses thereof [16]. The CAS number is 1253429-01-4 [18]. The application number is WO2010CN72224 20100427 and Priority number is CN2009183205 20090429. Yu investigated the syntheses and bioactivities of SYP-9625 and demonstrated its excellent acaricidal activity against *T*. *cinnabarinus* and its low acute toxicity to mammals.

### Selection of sublethal concentrations of SYP-9625

A modified leaf-residue method was used to determine the response of *T*. *cinnabarinus* to numerous concentrations of SYP-9625 which were based on initial range-finding test. Bean leaf disks (2 cm in diameter) were immersed for 5 s in solutions of SYP-9625 or a control (0.05% Tween 80 aqueous solution) and allowed to air dry. After eclosion, healthy *T*. *cinnabarinus* females were transferred onto the bean leaf disks. After 24 h, mites were separated onto untreated leaf disks to mate with males from the stock colony. Every 12 h, the fecundity of females was recorded until the females died naturally [3]. There were 30 individuals per replicate and four replicates per concentration.

A modified leaf dip method [19] was used to test the response of *T*. *cinnabarinus* eggs to the concentrations of SYP-9625 described above. 30 *N*. *californicus* female adults after coupled with males were placed on leaves for 12 h to allow oviposition and then were removed. Bean leaves with 50 eggs were then dipped for 5 s in solutions of SYP-9625 or a control (0.05% Tween 80 aqueous solution) and then placed upside down on a wet cotton pad soaked with distilled water. Eggs were checked daily and hatched in the laboratory. There were four replicates per concentration.

These two methods were also used for assessing the response of *N*. *californicus* females and eggs to the application concentration of SYP-9625 (100 μg/mL) and ten times the application concentration (1000 μg/mL).

### Experimental set up

All bioassays were carried out on primary bean leaf discs positioned upon moistened cotton wads in Petri dishes or tissue culture plates with the surface upward. Mites fear water, especially the predator mites. Therefore, water and the cotton soaked by water were used to prevent mites escape from bean leaf discs. This traditional method has been used in many other studies, including Alinejad M 2014 et al. and Hamedi N 2011 et al. [3, 5, 20].

**To assess the effects of sublethal concentrations of SYP-9625 on *T*. *cinnabarinus* and its offspring,** bean leaf disks (2 cm in diameter) were immersed for 10 s in sublethal concentrations (LC_10_ and LC_30_) or a 0.05% Tween 80 aqueous solution (control) and allowed to air dry. The subsequent processes were the same as those used for the selection of sublethal concentrations. Approximately 100 to 120 eggs were retained and transferred onto untreated bean leaf disks. The population parameters were recorded every 12 h after the eclosion for both sexes. The female offspring were mated with males from the stock colony and all indices were recorded until the females died naturally [3, 21].

**To assess the effects of the application concentration on *N*. *californicus* eggs**, a 3.5 cm diameter leaf disk with adequate quantities of *T*. *cinnabarinus* at each life stage as well as 30 *N*. *californicus* female adults after coupled with males were placed on leaves for 12 h to allow oviposition and then were removed. Bean leaves with 35 eggs were dipped in the solution with the application concentration (100 μg/mL) for 5 s and then placed upside down on a wet cotton pad soaked with distilled water. Eggs were checked daily and hatched in the laboratory. After hatching, the larvae were separated onto the untreated 2 cm diameter leaf disks using a 0.05% Tween -80 aqueous solution as a control. Population parameters were recorded every 12 h after eclosion for both sexes; all indices were recorded until all females died [22].

**The effects of the application concentration on *N*. *californicus* and its offspring from treated females were also tested.** The treatment method and setup were same as the experimental design for the determination of sublethal effects of SYP-9625 concentrations on *T*. *cinnabarinus* and its offspring from treated females.

**A modified method was conducted to assess the indirect effect on *N*. *californicus* and its offspring fed on *T*. *cinnabarinus* treated with sublethal concentrations of** SYP-9625. We fed *N*. *californicus* on treated females of *T*. *cinnabarinus* and evaluated the population parameters of predatory mites. Sufficient quantities of eggs of *N*. *californicus* fed on untreated *T*. *cinnabarinus* were collected over 24 h. When *N*. *californicus* grew to the deutonymph life stage, enough *T*. *cinnabarinus* females were treated with sublethal concentrations (LC_10_ and LC_30_) or with a 0.05% Tween-80 aqueous solution (control) using the same method as described above. After 24 h, *N*. *californicus* were fed on treated *T*. *cinnabarinus*, and *N*. *californicus* females were mated with males from the stock colony. Population parameters were then recorded every 12 h for both sexes and all indices were recorded until the females died.

There were 60 individuals of *N*. *californicus* per replicate and four replicates per concentration.

**To assess the effects of the application concentration on the functional response of *N*. *californicus***, bean leaf disks (4 cm in diameter) were immersed in the application concentration (100 μg/mL) or a 0.05% Tween-80 aqueous solution (control) and allowed to dry. Healthy *N*. *californicus* females were transferred onto the treated and untreated bean leaf disks within 12 h of copulation. After 24 h, they were individually transferred onto untreated bean leaf disks (1 cm×0.5 cm) and fed with *T*. *cinnabarinus* at each life stage. Egg and nymphal densities were 10, 15, 20, 25, and 30 per leaf. Larval densities were 10, 20, 30, 40, and 50 per leaf. Adult densities were 10, 15, 20, 25, and 30 per leaf. All leaves were placed in centrifuge tubes (2 ml) that were specially constructed to prevent mites from escaping [23].

**To assess the functional response of *N*. *californicus* fed on *T*. *cinnabarinus* treated with sublethal levels of SYP-9625,** healthy *N*. *californicus* females were individually introduced onto freshly cut leaf disks (2 mm×5 mm) that were placed in centrifuge tubes (0.5 ml) 12 h after copulation, starving for 24 h. *T*. *cinnabarinus* at all stages were treated for 24 h with sublethal concentrations (LC_10_ and LC_30_) of SYP-9625 or a 0.05% Tween -80 aqueous solution (control) using the same method described for determining the effect of sublethal concentrations on *T*. *cinnabarinus* and its offspring from treated females. *T cinnabarinus* were transferred onto leaf disks (1 cm×0.5 cm) with separately treated *N*. *californicus* using the same densities described above (see effects of the application concentration on the functional response of *N*. *californicus*).

There were five replicates per concentration. The functional response of *N*. *californicus* was observed and recorded after 24 h.

### Statistical analysis

The means and standard errors of the population parameters were estimated using a paired bootstrap test (TWOSEX-MS Chart) procedure [24] because it uses random resampling. The use of few replications can generate variable means and large standard errors (*P* < 0.05); thus, we used 10,000 replications.

The functional response of *N*. *californicus* to the various prey stages and densities were expressed by fitting Holling’s equation to the data [25–27]:

$$Na=\frac{aTN}{1+{aT}_{h}N}$$

Where *Na* is the number of prey attacked, *T* is the experimental time (1h), *N* is the initial number of prey offered, *a* is the searching (attack) rate, and *T_h_* is the handling time. Mean values of *T*_*h*_ were used to calculate the maximum attack rate defined as *T/Th*. The control efficiency of natural enemies can be represented by *a/Th*, as there is a positive correlation between *a/Th* and the control efficiency of natural enemies [28]. The searching rate, handling time and their asymptotic standard errors were estimated from nonlinear regressions of the disk equation. SAS statistical software was used to analyze the functional responses of *N*. *californicus*.

### Age-stage, two-sex life table

The raw data of the life table parameters were assessed with an age-stage, two-sex life table [29–34] using the computer program TWOSEX-MS Chart [35]. The age-stage specific survival rate (*s*_*xj*_) (where *x* = age in days and *j* = stage), female age-specific fecundity (*f*_*x5*_), age-specific survival rate (*l*_*x*_), age-specific fecundity (*m*_*x*_), *m*_*x*_ for the total population, age-specific maternity (*l*_*x*_*m*_*x*_) and the population growth parameters [the intrinsic rate of increase (*r*_*m*_), the finite rate of increase (*λ*), the net reproductive rate (*R*_*0*_), the gross reproductive rate (GRR), the mean generation time (*T*) and the doubling time (DT)] were calculated accordingly [3, 36].

## Results

### Determination of sublethal concentrations

The sublethal concentrations of SYP-9625 were chosen from the 24 h acute concentration-response relationship generated for adult females of *T*. *cinnabarinus* (Table 1) [9]. The LC_50_ of SYP-9625 on adult females and eggs were 0.466 μg/mL and 1.472μg/mL, respectively. The sublethal concentrations, including the LC_10_ (0.375μg/mL) and the LC_30_ (0.841μg/mL) were determined using a probit procedure (SAS Institute 2002) for the subsequent experiments and are summarized in Table 1. The regression equation of concentration-mortality for females was Y = 1.447+4.365X, [Y = mortality (probit), X = the log10 of concentration] (Table 1). No mortalities were recorded in the controls.

**Table 1:** Bioassay on different stages of *T*. *cinnabarinus* treated with SYP-9625.

Stage	LC-P line (y = )	Correlation coefficient(r)	*x*^2^	LC_50_/95%CL(μg/mL)	LC_30_/95%CL(μg/mL)	LC_10_/95%CL(μg/mL)
**Female**	1.447+4.365*x*	0.9945	3.219	0.466(0.442~0.492)	0.353(0.332~0.374)	0.237(0.216~0.256)
**Nymph**	5.311+10.994*x*	0.9975	0.537	0.329(0.316~0.341)	0.295(0.279~0.307)	0.251(0.232~0.267)
**Larva**	2.003+4.746*x*	0.9930	2.372	0.378(0.357~0.404)	0.293(0.275~0.331)	0.203(0.182~0.221)
**Egg**	-0.362+2.157*x*	0.9950	1.244	1.472(1.272~1.694)	0.841(0.688~0.990)	0.375(0.269~0.480)

The toxicity and field control efficacy of SYP-9625 to *T*. *urticae* has been tested by Gong et al [37]. Based on that study, an application concentration of 100μg/mL SYP-9625 was used in our experiment. After 24, 48 and 72 h per treatment, *N*. *californicus* females and eggs were both insensitive to the application concentration of SYP-9625. Even at ten times the application concentration, the hatching rate was 99.33±0.67. As a consequence, 100μg/mL of SYP-9625 was used as the application concentration on *N*. *californicus* in this study (Table 2).

**Table 2:** Effect of SYP-9625 on the survival rate of eggs and adult females of *N*. *californicus*.

Acaricide	Dose μg/mL	Hatching rate (%)	Survival rate (%)		
			24 h	48 h	72 h
**(SYP-9625)**	100	100.00±0.00a	100.00±0.00a	100.00±0.00a	99.67±0.33a
	1000	99.33±0.67a	100.00±0.00a	100.00±0.00a	99.00±0.58a
**Control**	/	99.67±0.33a	100.00±0.00a	100.00±0.00a	100.00±0.00a

Note: Data in the table are mean ± SE. Data in the same group followed by different letters indicate significant difference at the P<0.05 level using Duncan’s new multiple range test.

### Effects of sublethal concentrations of SYP-9625 on *T*. *cinnabarinus* females and their offspring

The Survival rate after 24 h treated by LC_30_ was 68%, which was significantly lower than the control (100%). The total spawning rate, female longevity and the fecundity of *T*. *cinnabarinus* females treated with sublethal concentrations (LC_10_, LC_30_) were significantly reduced, and the pre-oviposition periods were significantly extended compared with the controls (Table 3). The oviposition period of females in the LC_30_ treatment was significantly shorter than oviposition period of females in the control treatment. The total spawning rate, female longevity and fecundity of females in the LC_30_ treatment were lower than females exposed to the LC_10_ treatment. Moreover, the pre-oviposition periods in the LC_30_ treatment were longer than in the LC_10_ treatment.

**Table 3:** Effects of sublethal exposure to SYP-9625 on the fecundity and longevity of treated females of *Tetranychus cinnabarinus*.

Parameter	Control	SYP-9625	
		LC_10_	LC_30_
**Survival rate after 24h (%)**	100	92	68
**Total spawning rate (%)**	100.00±0.00a	72.13±7.48b	40.48±7.19cd
**Preoviposition period (days±*SE*)**	1.71±0.07d	2.75±0.07c	3.42±0.11a
**Oviposition period (days±*SE*)**	6.00±0.52a	4.85±0.61ab	4.10±0.65b
**Fecundity per female (eggs±*SE*)**	26.06±2.75a	12.53±2.29b	5.40±1.43cd
**Female longevity (days±*SE*)**	9.14±0.86a	6.89±0.70b	4.44±0.56cd

Note: The total spawning rate is the number of spawning individuals/ the total number of individuals that are effectively processed.

Data in the table are mean ± SE. Data in the same group followed by different letters indicate significant difference at the P<0.05 level using Duncan’s new multiple range test.

**Fig 1:**
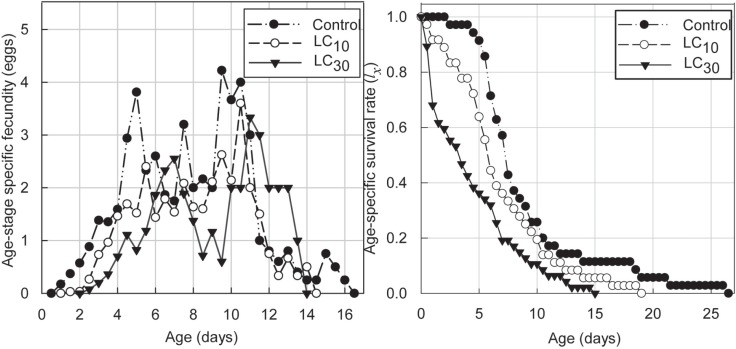
Female age-specific fecundity (*fx*_5_) and the age-specific survival rate (*lx*) of female adults *T*. *cinnabarinus* treated with sublethal concentrations of SYP-9625.

Fig 1 shows that the age-specific fecundity curves and the peak values of adult females *T*. *cinnabarinus* treated with sublethal concentrations (LC_10_, LC_30_) of SYP-9625 shifted. Moreover, a significant reduction in the age-specific survival rate was observed at both concentrations.

**Fig 2:**
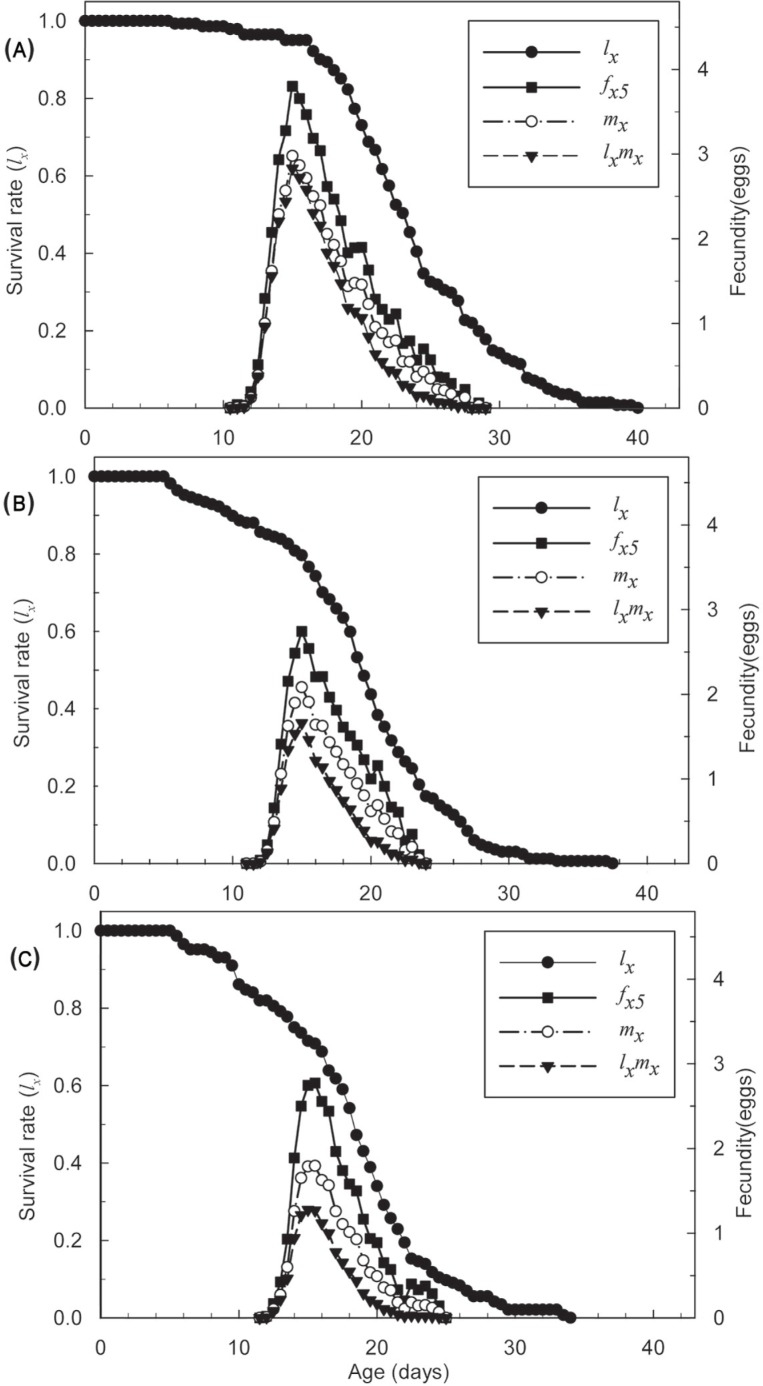
Age-specific survival rate (*l*_*x*_), female age-specific fecundity (*f*_*x5*_), age-specific fecundity of the total population (*m*_*x*_), and age-specific maternity (*l*_*x*_*m*_*x*_) of *T*. *cinnabarinus* eggs treated with sublethal concentrations of SYP-9625. (A) Control, (B) LC_10_, (C) LC_30_.

Moreover, the total survival rate was lowerin the LC_30_ treatment than in the LC_10_ treatment and the control. In Fig 2, the slope of *l*_*x*_ increased after 5 to 16 days as the sublethal concentration increased from LC_10_ to LC_30_, but they converged on the same value. The peak values of *f*_*x5*_ and *l*_*x*_*m*_*x*_ in individuals that survived the LC_10_ and LC_30_ treatments were distinctly lower than in the control, but less difference was observed between the LC_10_ and LC_30_ treatments. Consequently, sublethal concentrations of SYP-9625 weakened reproduction in the population, particularly the fecundity of female mites.

As shown in Table 4, the *r*_*m*_, *λ* and *R*_*0*_ of offspring from treated *T*. *cinnabarinus* females were significantly lower than the control. The increasing concentration produced a dramatic change. Additionally, the *T* in the LC_30_ treatment was significantly shorter than in the control.

**Table 4:** Population life table parameters for offspring from females of *Tetranychus cinnabarinus* treated with sublethal concentrations of SYP-9625.

Parameter	Control	SYP-9625	
		LC_10_	LC_30_
**Intrinsic rate of increase rate, *r***_***m***_ **(d**^**-1**^**)**	0.209±0.003a	0.166±0.005b	0.147±0.006c
**Finite rate of increase, *λ* (d**^**-1**^**)**	1.232±0.004a	1.180±0.006b	1.158±0.007c
**Net reproductive rate, *R***_***0***_ **(offspring/individual)**	32.74±2.03a	14.96±1.23b	11.02±1.17c
**Mean generation time, *T* (d)**	16.72±0.09a	16.33±0.11a	16.33±0.13a

Note: Data in the table are mean ± SE. Data in the same group followed by different letters indicate significant difference at the P<0.05 level using Duncan’s new multiple range test.

### Effects of the application concentration of SYP-9625 on *N*. *californicus* eggs

After a 5 s exposure to the application concentration (100μg/mL), preadult duration, longevity and the total life span of adults from the treated eggs of *N*. *californicus* were not significantly influenced, as shown in Table 5. Larval and protonymph durations in treatment groups were longer than the control. Beyond that, other indices including female proportion and the adult emergence rate showed less difference with the control. Table 6 presents the spawning rate, pre-oviposition and fecundity per female among the females grown from treated eggs. The total duration of pre-oviposition for females from eggs treated with SYP-962 was significantly longer than the control; in contrast, the duration of oviposition was shorter.

**Table 5:** Development time, longevity, and total life span of *Neoseiulus californicus* eggs treated with the application concentration of SYP-9625.

Parameter	Control	SYP-9625(100μg/mL)
**Female proportion (%)**	62.88±4.91a	60.99±4.87a
**Adult emergence rate (%)**	97.00±1.70ab	100.00±0.00a
**Female**		
**Egg duration (d)**	1.83±0.07a	1.84±0.03a
**Larva duration (d)**	0.58±0.03b	0.73 ± 0.03a
**Protonymph duration (d)**	0.98± 0.01b	1.05 ±0.02a
**Deutonymph duration (d)**	1.22±0.06a	1.27±0.25a
**Preadult duration (d)**	4.61±0.04b	4.89± 0.04a
**Longevity (d)**	30.95±1.19a	26.30±1.37b
**Total life span (d)**	35.56±1.21a	31.18±1.38b
**Male**		
**Egg duration (d)**	1.86± 0.04a	1.91± 0.03a
**Larva duration (d)**	0.58± 0.03a	0.59± 0.03a
**Protonymph duration (d)**	0.88±0.04b	0.91±0.03b
**Deutonymph duration (d)**	1.01±0.03a	1.08±0.03a
**Preadult duration (d)**	4.33±0.07b	4.49±0.07ab
**Longevity (d)**	33.04±2.22a	29.58±2.06a
**Total life span (d)**	37.38±2.25a	34.06±2.05a

Note: Data in the table are mean ± SE. Data in the same group followed by different letters indicate significant difference at the P<0.05 level using Duncan’s new multiple range test.

**Table 6:** The reproduction and fecundity of *Neoseiulus californicus* eggs treated with the application concentration of SYP-9625.

Parameter	Control	SYP-9625(100μg/mL)
**Spawning rate (%)**	100.00±0.00a	100.00±0.00a
**Pre-oviposition (d)**	1.68±0.04a	1.72±0.05a
**Total pre-oviposition (d)**	6.29±0.07b	6.61±0.08a
**Oviposition (d)**	15.77±0.44a	13.30±0.45b
**Fecundity per female (eggs)**	46.72±1.35a	40.26±1.42ab

Note: Data in the table are mean ± SE. Data in the same group followed by different letters indicate significant difference at the P<0.05 level using Duncan’s new multiple range test.

**Fig 3:**
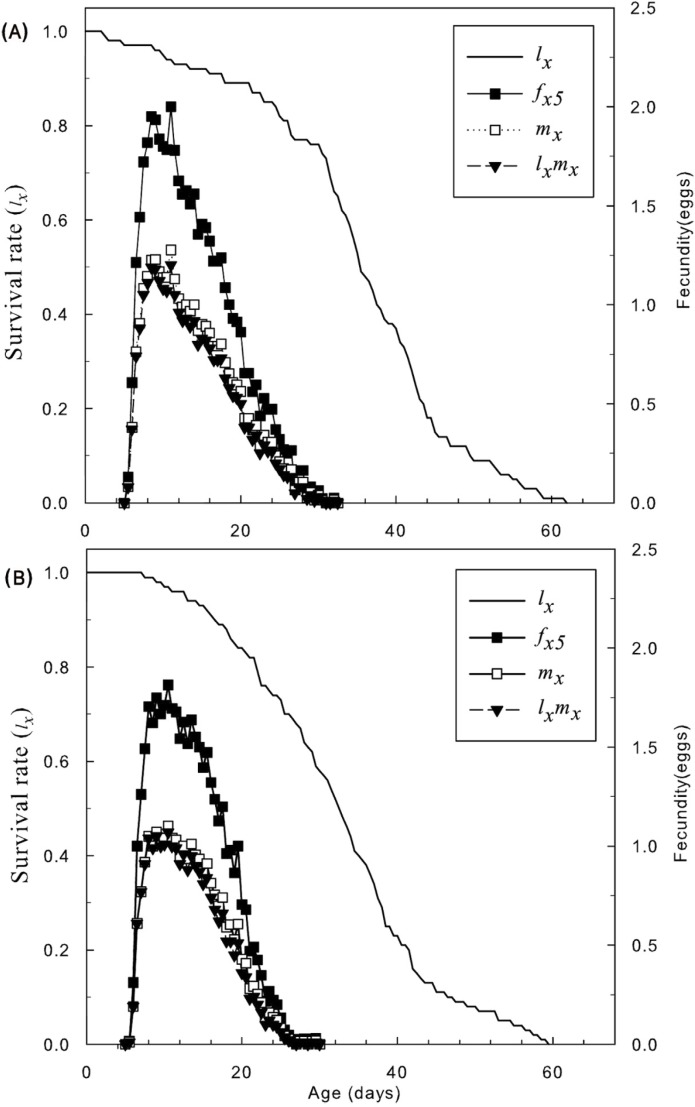
Age-specific survival rate (*l*_*x*_), female age-specific fecundity (*f*_*x5*_), age-specific fecundity of the total population (*m*_*x*_), and age-specific maternity (*l*_*x*_*m*_*x*_) of *N*. *californicus* (McGregor) eggs treated with sublethal concentrations of SYP-9625. (A) Control, (B) SYP-9625.

The difference in *l*_*x*_, *f*_*x5*_ and *m*_*x*_ in the total population between treatments and control could barely been distinguish. The peak value of *f*_*x5*_ for the control (2) occurred at 11 days, and the peak value of *f*_*x5*_ 1.8 for the application concentration occurred at 10 days in Fig 3. The *r*_*m*_, *λ*, GRR and *T* of treated *N*. *californicus* eggs were not significantly different from the control (Table 7). Hence, there was little effect on the population growth of *N*. *californicus* eggs exposed to the application concentration of SYP-9625.

**Table 7:** Population life table parameters of *Neoseiulus californicus* eggs treated with the application concentration of SYP-9625.

Parameter	Control	SYP-9625(100μg/mL)
**Intrinsic rate of increase rate, *r***_***m***_ **(d**^**-1**^**)**	0.292±0.009a	0.277±0.009a
**Finite rate of increase, *λ* (d**^**-1**^**)**	1.338±0.012a	1.319±0.012a
**Net reproductive rate, *R***_***0***_ **(offspring/individual)**	28.50±2.41a	24.56±2.15ab
**Mean generation time, *T* (d)**	11.49±0.14a	11.55±0.16a

Note: Data in the table are mean ± SE. Data in the same group followed by different letters indicate significant difference at the P<0.05 level using Duncan’s new multiple range test.

### Effects of the application concentration of SYP-9625 on *N*. *californicus* females and their offspring

**Fig 4:**
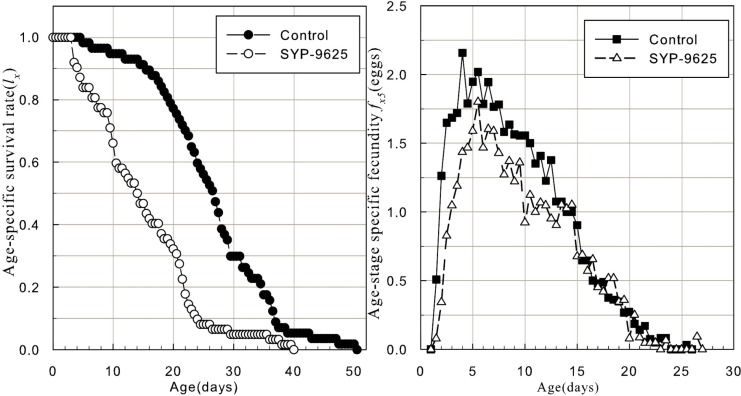
Age-specific survival rate (*l*_*x*_) and female age-specific fecundity (*f*_*x5*_) of *N*. *californicus* (McGregor) adult females treated with sublethal concentrations of SYP-9625.

The application concentration reduced the survival rate of treated females (Fig 4). The peak value of female age-specific fecundity occurred earlier in the control than in the treatment. Additionally, the fluctuation in female age-specific fecundity was greater than in the control.

**Fig 5:**
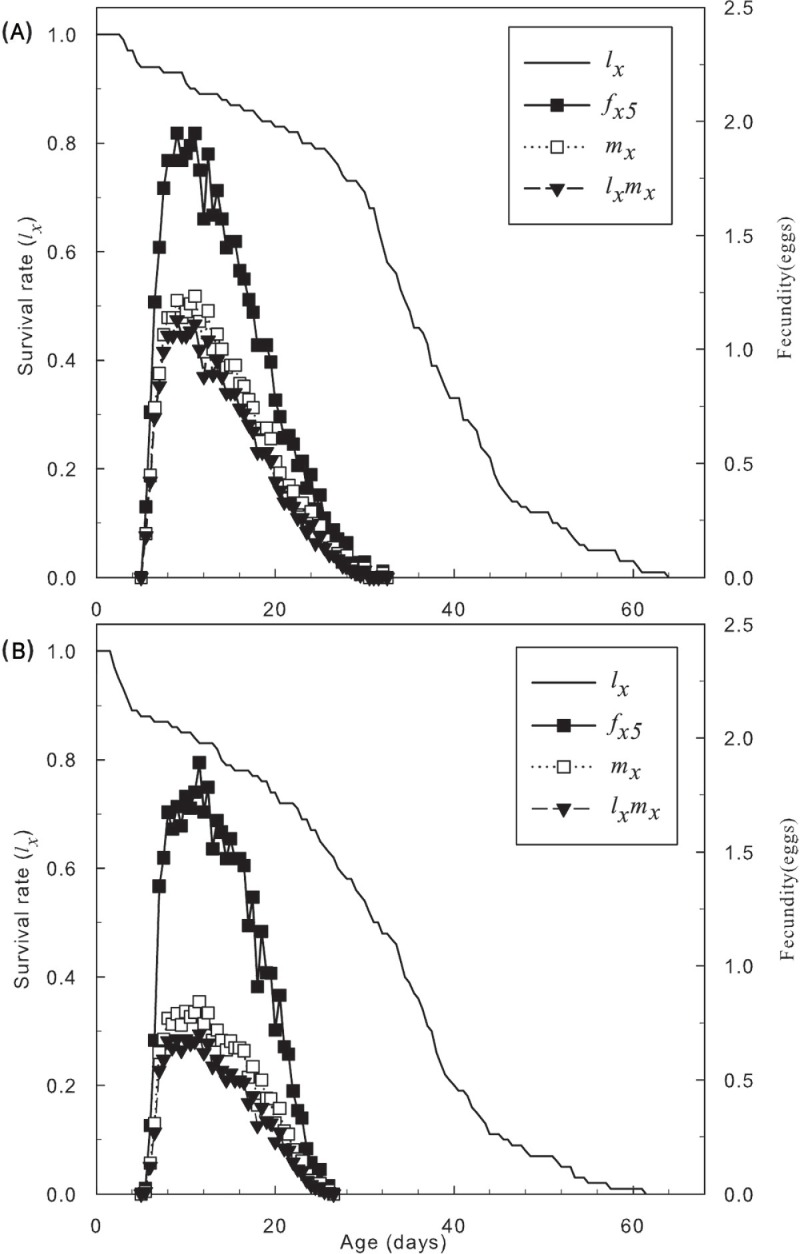
Age-specific survival rate (*l*_*x*_), female age-specific fecundity (*f*_*x5*_), age-specific fecundity of the total population (*m*_*x*_), and age-specific maternity (*l*_*x*_*m*_*x*_) of offspring from adult female *N*.*californicus* (McGregor) treated with sublethal concentrations of SYP-9625. (A) Control, (B) SYP-9625.

Initially, the age-specific survival rate at the application concentration declined slowly from 0 d to 30 d. Age-specific survival rate then decreased more rapidly from 30 d to 60 d. The acaricide treatment barely affected the age-specific survival rate of offspring from treated females of *N*. *californicus*, and the declining gradient of the earlier stage was higher than the control in Fig 5.

Compared with the control, the *R*_*0*_, *r*_m_ and *λ* of offspring from individual *N*. *californicus* females treated with SYP-9625 were significantly lower (Table 8). However, there was no significant difference in the *T* between the treatment and control.

**Table 8:** Population life table parameters of offspring from *Neoseiulus californicus* females treated with the application concentration of SYP-9625.

Parameter	Control	SYP-9625(100μg/mL)
**Intrinsic rate of increase rate, *r***_***m***_ **(d**^**-1**^**)**	0.290±0.009a	0.233±0.012b
**Finite rate of increase, *λ* (d**^**-1**^**)**	1.336±0.012a	1.263±0.155b
**Net reproductive rate, *R***_***0***_ **(offspring/individual)**	27.37±2.43a	15.91±2.08b
**Mean generation time, *T* (d)**	11.42±0.14a	11.87±0.19a

Note: Data in the table are mean ± SE. Data in the same group followed by different letters indicate significant difference at the P<0.05 level using Duncan’s new multiple range test.

### Effects on *N*. *californicus* fed on *T*. *cinnabarinus* treated with sublethal levels of SYP-9625

**Fig 6:**
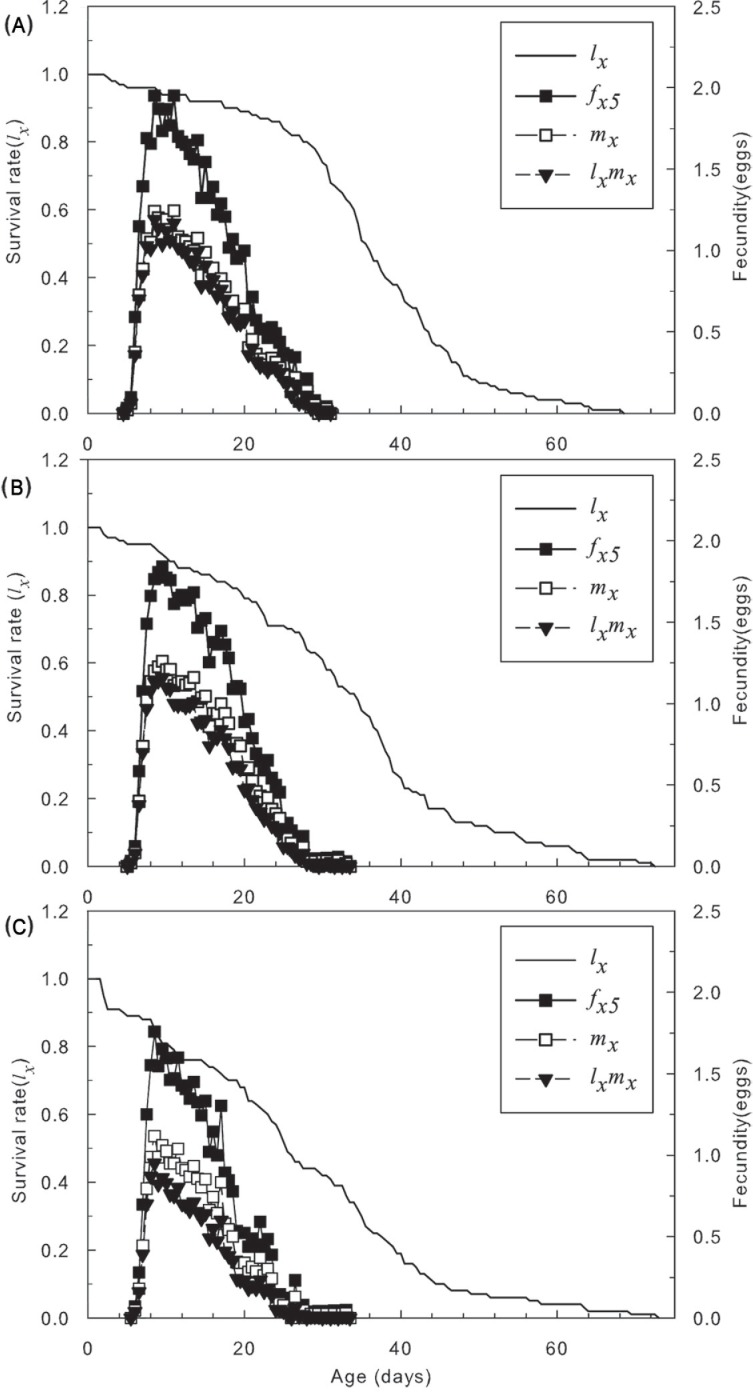
Age-stage specific survival rate (*s*_*xj*_) of offspring from adult female *N*. *californicus* (McGregor) fed on *T*. *cinnabarinus* treated with sublethal concentrations of SYP-9625. (A) Control, (B) LC_10_, (C) LC_30_.

As shown in Fig 6, *l*_*x*_ rapidly declined between 0 to 30 d with increased concentrations of SYP-9625; *l*_*x*_ declined more slowly from 30 to 48 d. After 48 d, all *l*_*x*_ values gradually decreased to 0% between 64.5 to 72.5 d. The *fx*_5_, *m*_x_ and *l*_x_*m*_x_ for the LC_10_ treatment were not significantly different from the control, but the *fx*_5,_ *m*_x_ and *l*_x_*m*_x_ for the LC_30_ treatment were all lower than the control. All of the population parameters for the LC_30_ treatment were lower than the control, with the exception of *T* (Table 9). After *N*. *californicus* were fed on treated *T*. *cinnabarinus*, the *r*_*m*_ of the subsequent generation was significantly reduced from 0.289 to 0.243. The intrinsic rate of increase rate (*r*_*m*_) is an important parameter affecting variation in the population trend under specific environmental conditions and reflects the reproductive capacity of *N*. *californicus*. Additionally, *λ* was significantly reduced from 1.335 to 1.275, and *R*_*0*_ was significantly reduced from 28.71 to 18.13. However, the population parameters of the LC_10_ treatment were similar to those of the control.

**Table 9:** Population life table parameters of offspring from *Neoseiulus californicus* fed on *Tetranychus cinnabarinus* treated with sublethal levels of SYP-9625.

Parameter	Control	SYP-9625	
		LC_10_	LC_30_
**Intrinsic rate of increase rate, *r***_***m***_ **(d**^**-1**^**)**	0.289±0.009a	0.278±0.008a	0.243±0.009b
**Finite rate of increase, *λ* (d**^**-1**^**)**	1.335±0.012a	1.319±0.010a	1.275±0.012b
**Net reproductive rate, *R***_***0***_ **(offspring/individual)**	28.71±2.51a	28.02±2.37a	18.13±1.85b
**Mean generation time, *T* (d)**	11.61±0.15a	12.01±0.15a	11.91±0.16a

Note: Data in the table are mean ± SE. Data in the same group followed by different letters indicate significant difference at the P<0.05 level using Duncan’s new multiple range test.

### Effects of the application concentration on control efficiency of *N*. *californicus*

The control efficiency of *N*. *californicus* had an intrinsic acceleration owing to inverse density-dependent effects after adult female *N*. *californicus* were treated with 100 μg/mL of SYP-9625. There was no significant effect on the daily consumption of *N*. *californicus* against eggs and densities of 5, 10, or 15 adults of *T*. *cinnabarinus* per leaf. The daily consumption of nymphs significantly differed at densities of 10, 15, 20 and 30 nymphs per leaf. The daily consumption of larvae and adults declined significantly at densities of 30 and 50 per leaf and 20 and 25 per leaf, respectively (Table 10).

**Table 10:** Daily consumption by *Neoseiulus californicus* exposed to the application concentration of SYP-9625.

**Stages of preys**	Treatments	Density of *Tetranychus cinnabarinus* (number per leaf)							
		5	10	15	20	25	30	40	50
**Egg**	CK	—	7.60±0.68a	10.27±0.66b	12.00±0.63ab	15.00±0.63a	15.27±0.49a	—	—
	SYP-9625	—	8.00±0.63a	13.50±0.22a	14.50±0.22a	14.50±0.50a	14.50±0.22ab	—	—
**Larva**	CK	—	10.00±0.00a	—	19.00±0.55a	—	28.60±0.75a	30.60±0.81ab	31.00±0.63b
	SYP-9625	—	10.00±0.00a	—	19.00±0.32a	—	26.10±0.56b	29.00±0.32b	28.50±0.81c
**Nymph**	CK	—	10.00±0.00a	12.60±0.81b	19.20±0.20b	19.60±1.33a	20.00±0.32b	—	—
	SYP-9625	—	9.50±0.22b	14.50±0.22a	20.00±0.00a	18.00±0.32a	24.50±1.75a	—	—
**Adult**	CK	3.40±0.24b	4.00±0.32b	5.20±0.37b	6.60±0.40a	8.40±0.24a	—	—	—
	SYP-9625	3.50±0.22b	4.00±0.00b	4.00±0.32b	5.50±0.22b	7.50±0.22b	—	—	—

Note: Data in the table are means ± SE. Data in the same group (column and life stage) followed by different letters indicate a difference at the P < 0.05 level using by Duncan’s new multiple range test. “-” indicates that the treatments of corresponding densities were not processed.

The functional response of *N*. *californicus* fits reasonably well to a type II functional response of the Holling model (Table 11). The application concentration led to a reduction in handling time and attack rate against the different life stages, with the exception of nymphs. Compared with the control, the maximum attack rates *(T/T*_*h*_*)* of *N*. *californicus* against nymphs was 128.2051, which was the highest value among the different stages. The control efficiency (*a/T*_*h*_) of eggs and nymphs increased by 27.39% and 74.54%, respectively. *a/T*_*h*_ of larvae and adults decreased by 19.71% and 18.98%, respectively.

**Table 11:** Functional response models and parameters of *Neoseiulus californicus* exposed to the application concentration of SYP-9625.

Stage of prey	Treatment	Functional response equation	Correlation coefficient	Attack rate (*a*)	Handling time (*T*_*h*_)	*T/T*_*h*_	*a/T*_*h*_
**Egg**	CK	*Na* = 0.9746*N*/(1+0.0283*N*)	0.9801	0.9746	0.0290	34.4828	33.6069
	SYP-9625	*Na* = 1.3959*N*/(1+0.0505*N*)	0.9207	1.3959	0.0326	30.6748	42.8190
**Larva**	CK	*Na* = 1.1614*N*/(1+0.0127*N*)	0.9257	1.1614	0.0109	91.7431	106.5505
	SYP-9625	*Na* = 1.2190*N*/(1+0.0174*N*)	0.9170	1.2190	0.0143	69.9301	85.2448
**Nymph**	CK	*Na* = 1.1272*N*/(1+0.0168*N*)	0.9148	1.1272	0.0149	67.1141	75.6510
	SYP-9625	*Na* = 1.0299*N*/(1+0.0081*N*)	0.9353	1.0299	0.0078	128.2051	132.0385
**Adult**	CK	*Na* = 0.7020*N*/(1+0.0514*N*)	0.9904	0.7020	0.0732	13.6612	9.5902
	SYP-9625	*Na* = 0.6791*N*/(1+0.0594*N*)	0.9184	0.6791	0.0874	11.4416	7.7700

### Control efficiency of *N*. *californicus* fed on *T*. *cinnabarinus* treated with sublethal acaricide

The functional response model parameters for *N*. *californicus* fed on *T*. *cinnabarinus* treated with sublethal acaricide were altered for various life stages (Table 12). There was a significant increase in the daily consumption of *N*. *californicus* against eggs at densities of 10, 15 and 20 *T*. *cinnabarinus* eggs per leaf with an increased concentration of SYP-9625. There was no significant difference in the control efficiency against nymphs among all treatments and the control at densities of 10, 15, or 20 nymphs per leaf. When the nymphal density increased to 25 and 30 per leaf, the daily consumption was higher in the treatments than in the control. There was little difference in control efficiency among all treatments and the control at densities of 5, 10 and 15 adults per leaf. When the adult density increased to 20 and 25 per leaf, the daily consumptions were significantly lower than the control.

**Table 12:** Daily consumption of *Neoseiulus californicus* fed on *Tetranychus cinnabarinus* treated with sublethal acaricide.

Stage of prey	Treatment	Density of *Tetranychus cinnabarinus* (number per leaf)							
		5	10	15	20	25	30	40	50
**Egg**	CK	—	7.60±0.68b	10.27±0.66b	12.00±0.63b	15.00±0.63a	15.27±0.48a	—	—
	LC_10_	—	9.40±0.24a	12.40±0.24a	14.20±0.20a	14.50±0.22a	15.00±0.00a	—	—
	LC_30_	—	8.00±0.63ab	12.00±0.32a	15.50±0.50a	15.50±0.22a	15.60±0.24a	—	—
**Larva**	CK	—	10.00±0.00a	—	19.00±0.55a	—	28.60±0.75a	30.60±0.81a	31.00±0.63a
	LC_10_	—	10.00±0.00a	—	20.00±0.00a	—	29.33±0.18a	28.33±0.66b	32.67±0.80a
	LC_30_	—	10.00±0.00a	—	19.33±0.37a	—	29.33±0.18a	29.67±0.48ab	31.00±0.84a
**Nymph**	CK	—	10.00±0.00a	12.60±0.81a	19.20±0.20a	19.60±1.33b	20.00±0.32b	—	—
	LC_10_	—	10.00±0.00a	13.60±0.40a	18.40±0.40a	21.60±0.24ab	22.00±0.55a	—	—
	LC_30_	—	10.00±0.00a	12.67±0.18a	18.33±0.18a	22.27±0.19a	22.67±0.18a	—	—
**Adult**	CK	3.40±0.24a	4.00±0.32a	5.20±0.37a	6.60±0.40a	8.40±0.24a	—	—	—
	LC_10_	4.00±0.32a	4.400±0.24a	4.60±0.24a	5.00±0.32b	5.00±0.32b	—	—	—
	LC_30_	4.00±0.00a	4.33±0.18a	4.33±0.37a	5.00±0.55b	5.33±0.18b	—	—	—

Note: Data in the table are means ± SE. Data in the same group (column and life stage) followed by different letters indicate a difference at the P < 0.05 level using by Duncan’s new multiple range test. “-” indicates that the treatments of corresponding densities were not processed.

The functional response model fits reasonably well to a type II functional response of the Holling model based on the parameters in Table 13. The sublethal concentrations led to an increase in the attack rate against all life stages compared with the control. The attack rates against adults in the LC_10_ and LC_30_ treatments increased by 344.64% and 176.71%, respectively. The handling time of the different life stages did not differ at any concentration, except that the handling time of adults was longer than the control. The highest value of *T/T*_*h*_ was 107.5269 against nymphs in the LC_30_ treatment, which was the maximum attack rate documented in this experiment. The maximum *a/T*_*h*_ (112.9677) was also observed for nymphs in the LC_30_ treatment. However, the *a/T*_*h*_ against adults had a maximum value at LC_10_. When the concentration of SYP-9625 reached the LC_30_, the value of *a/T*_*h*_ was still higher than the control, but a decrease was observed.

**Table 13:** Functional response model and parameters of *Neoseiulus californicus* fed on *Tetranychus cinnabarinus* treated with sublethal acaricide.

Stage of prey	Treatment	Functional response equation	Correlation coefficient	Attack rate (*a*)	Handling time (*T*_*h*_)	*T/T*_*h*_	*a/T*_*h*_
**Egg**	CK	*Na* = 0.9746*N*/(1+0.0283*N*)	0.9801	0.9746	0.0290	34.4828	33.6069
	LC_10_	*Na* = 1.7287*N*/(1+0.0776*N*)	0.9262	1.7287	0.0449	22.2717	38.5011
	LC_30_	*Na* = 1.0281*N*/(1+0.0241*N*)	0.9024	1.0281	0.0234	42.7350	43.9359
**Larva**	CK	*Na* = 1.1614*N*/(1+0.0127*N*)	0.9257	1.1614	0.0109	91.7431	106.5505
	LC_10_	*Na* = 1.1805N/(1+0.0130*N*)	0.9282	1.1805	0.0110	90.9091	107.3182
	LC_30_	*Na* = 1.1660*N*/(1+0.0128*N*)	0.9148	1.1660	0.0110	90.9091	106.0000
**Nymph**	CK	*Na* = 1.1272*N*/(1+0.0168*N*)	0.9148	1.1272	0.0149	67.1141	75.6510
	LC_10_	*Na* = 1.1529*N*/(1+0.0156*N*)	0.9711	1.1529	0.0135	74.0741	85.4000
	LC_30_	*Na* = 1.0506*N*/(1+0.0098*N*)	0.9711	1.0506	0.0093	107.5269	112.9677
**Adult**	CK	*Na* = 0.7020*N*/(1+0.05136*N*)	0.9904	0.7020	0.0732	13.6612	9.5902
	LC_10_	*Na =* 3.1214*N*/(1+0.1926*N*)	0.9707	3.1214	0.1926	5.1921	16.2066
	LC_30_	*Na* = 1.9923*N*/(1+0.3468*N*)	0.9623	1.9923	0.1741	5.7438	11.4434

## Discussion

In previous studies, many species of natural enemies and pesticides have been tested so far to corroborate the combination of chemical and biological control agents under laboratory conditions [38–42]. Moreover, numerous studies have focused on the importance of sublethal effects of pesticides on predatory mites [3, 9, 33]. On one hand, this is the first report on both pest mites and the predatory mites of the new pesticide SYP-9625. On the other hand, *N*. *californicus* provides good efficacy against pest mites as showed by most studies [15, 43]. Therefore, this study was designed to examine the appropriate concentration of SYP-9625 that can be used to control the increasing population of *T*. *cinnabarinus* effectively and simultaneously protect *N*. *californicus*.

### Sublethal effects of SYP-9625 on *T*. *cinnabarinus*

**Our results showed that the sublethal concentration of SYP-9625 can effectively inhibit the increasing population of *T*. *cinnabarinus*.** The overall impact on *T*. *cinnabarinus* offspring is greater for females than for males, which was approximately similar to the results obtained by Asma et al. for *T*. *urticae* treated with a series of biopesticide concentrations (0.31-10ml/l) [20]. The population parameters (*r*_*m*_, *λ* and *R*_*0*_) of offspring treated with sublethal concentrations decreased significantly as the concentration increased, which is consistent with the findings of Asma et al. and Dejan [20, 44].

### Effects of SYP-9625 on *N*. *californicus*

Our results revealed that the application concentration negatively affected the survivorship of *N*. *californicus* adulthood and its subsequent generation, which is consistent with the findings of Maryam et al for *N*. *californicus* treated with LC_15_ sublethal concentration of spiromesifen [45]. In addition, the *r*_*m*_, *λ* and *R*_*0*_ of offspring from *N*. *californicus* females fed on *T*. *cinnabarinus* treated with an LC_30_ of SYP-9625 were significantly reduced, which is partly consistent with the previous findings [4]. Many indices of *N*. *californicus* eggs exposed to the application concentration (100μg/mL) preadult duration, longevity, total life span, female proportion and adult emergence rate showed less difference when compared with the control. All the results showed that the application concentration of SYP-9625 had little influence on the development and fecundity of *N*. *californicus* eggs. This demonstrates that *N*. *californicus* eggs were able to tolerate the application concentration of SYP-9625 (100 mg/L).

### Effects of SYP-9625 on the functional response of *N*. *californicus*

*N*. *californicus* exhibited a Holling type-II type functional response when fed on *T*. *cinnabarinus* exposed to sublethal concentrations of SYP-9625, and no changes in the functional response model were observed. Similarly, Li et al. showed that a Holling type—II functional response was exhibited by predatory thrips *Scolothrips*. *takahashii* fed on *Tetranychus viennensis* except for female [46]. The attack rate of *N*. *californicus* expoed to the application concentration of SYP-9625 increased compared with the control, except for the attack rate on nymphs treatment. The attack rate against treated *T*. *cinnabarinus* increased as well, particularly for adults. In contrast, Angeliki et al. reported that sublethal concentrations of thiacloprid led to a significant reduction of the attack rate of *Macrolophus pygmaeu*s [28]. In general, most of the handling time (*T*_*h*_) of *N*. *californicus* against treated *T*. *cinnabarinus* and the handling time of *N*. *californicus* exposed to the application concentration was longer than the control, which is consistent with the study on *M*. *pygmaeus* exposed to thiacloprid and chlorantraniliprole [28]. The control efficiency *a/T*_*h*_ against treated adult *T*. *cinnabarinus* reached a maximum value in the LC_10_ treatment. Furthermore, the *a/Th* against the larval and nymphal stages were significantly higher than other stages. Consequently, the predation ability of *N*. *californicus* against sublethal treated *T*. *cinnabarinus* and the predation ability of *N*. *californicus* exposed to the application concentration were both significantly positively affected, particularly at the lower sublethal concentration of SYP-9625 (LC_10_). This result differed from other studies such as Rashidi et al. which found that sublethal doses of four pesticides negatively affected the control efficiency of *Habrobracon*. *Hebetor* [47]. It might due to the weak toxicity of SYP-9625 against *N*. *californicus*, and a hormesis effect at lower concentrations (LC_10_) stimulates the trophic behavior of *N*. *californicus*. It is reported that the hormesis effect occurs at a low doses in a number of ecological populations such as the control efficiency of *Pardosa agrestis* treated with eight herbicides and *Supputius cincticeps* treated with sublethal concentrations of permethrin [48, 49].

We maintain that a lower concentration (LC_10_ = 0.375 μg/mL) of SYP-9625 is beneficial for *N*. *californicus*. SYP-9625 at the LC_10_ can stimulate the predation capability against *T*. *cinnabarinus* and is also safe for *N*. *californicus* eggs.

## Conclusions

The sublethal effects of SYP-9625 on *T*. *cinnabarinus*, the effects of application concentration of SYP-9625 on the predatory mite *N*. *californicus* and the functional response of *N*. *californicus* were successfully assessed. This study concludes that SYP-9625, particularly at a lower concentration (LC_10_ = 0.375 μg/mL) can effectively control the increasing population of *T*. *cinnabarinus* and stimulate the predation capability of *N*. *californicus*. We confirmed that the new acaricide SYP-9625 can be used in concert with the release of the predator *N*. *californicus* in IPM.

## Supporting information

S1 File The structure of SYP-9625. (EPS)
